# Enhancing the rational use of sodium valproate in neurosurgery: a pharmacist-led PDCA intervention

**DOI:** 10.3389/fphar.2026.1786783

**Published:** 2026-03-09

**Authors:** Zhuoxue Li, Yahao Ling, Guifang Rao, Mengkao Li, Zhili Zhao, Li Gong, Shiqin Dai, Tao Shi

**Affiliations:** 1 Department of Pharmacy, People’s Hospital of Longhua, Shenzhen, China; 2 Department of Neurosurgery, People’s Hospital of Longhua, Shenzhen, China

**Keywords:** defined daily dose (DDD), neurosurgery, PDCA cycle (plan-do-check-act), pharmacist-led intervention, prescription evaluation, rational drug use, sodium valproate

## Abstract

**Background and Objective:**

Inappropriate use of sodium valproate (VPA) for seizure prophylaxis in neurosurgery poses clinical risks and increases healthcare costs. This study aimed to evaluate the effectiveness of a clinical pharmacist-led intervention, integrated with the Plan-Do-Check-Act (PDCA) cycle, in optimizing VPA utilization among neurosurgical patients.

**Methods:**

This retrospective pre-post study analyzed patients in the Neurosurgery Department of a tertiary hospital who received sodium valproate between July 2022 and December 2024. A multidisciplinary team, led by clinical pharmacists, implemented a PDCA cycle to standardize sodium valproate administration. Key metrics—including irrational drug use, treatment duration, and Defined Daily Doses (DDDs)—were compared pre- and post-implementation.

**Results:**

The intervention significantly increased the rational use of sodium valproate from 32.00% to 93.41%. This clinical shift was accompanied by substantial resource optimization, including a reduction in the average duration of injectable therapy (from 8.32 ± 6.44 to 5.37 ± 3.81 days) and a dramatic decline in the DDDs of prophylactic intravenous sodium valproate (from 184.69 ± 50.40 to 17.91 ± 8.92). Furthermore, inappropriate average DDDs were reduced from 0.55 ± 0.22 to 0.17 ± 0.09.

**Conclusion:**

A pharmacist-led PDCA cycle is a feasible and highly effective strategy for fostering the rational use of sodium valproate in neurosurgical settings. These findings highlight the value of multidisciplinary frameworks in enhancing medication safety and institutional resource management within complex clinical environments.

## Highlights

• A pragmatic strategy for implementing Plan-Do-Check-Act (PDCA) tools into regular tasks using a multidisciplinary approach.
• Clinical pharmacist-led stewardship enhanced the appropriate use of sodium valproate (VPA).
• The retrospective pre-post study design may limit the generalizability of these findings to other healthcare settings.

## 1 Introduction

Seizures are a commonly encountered complication following acute neurological insults, such as traumatic brain injury (TBI), and intracerebral hemorrhage (ICH) (Fordington and Manford, 2020; Pease et al., 2022; Derex et al., 2021). In adults, the risk of developing seizures is significantly higher, with severe TBI cases showing a cumulative incidence of 25% at 5 years and 32% at 15 years post-injury (Pease et al., 2022). Similarly, post-ICH seizures affect 6%–15% of patients, with the incidence rising to 30% when including subclinical seizures detected by Electroencephalogram (Derex et al., 2021). These seizures not only increase the immediate risk but also heighten the probabilities of intracranial hemorrhage, intracranial edema, leading to longer hospital stays and higher healthcare cost (Garg et al., 2021).

Sodium valproate (VPA), one of the most commonly used anti-seizure medications (ASMs) in clinical practice, is being explored as a method to decrease the incidence of epileptic seizures in this population (Marson et al., 2021). However, its utility remains controversial due to the lack of strong evidence supporting its efficacy and concerns about potential adverse effects (Wat et al., 2019; Mota Telles et al., 2023). Issues regarding the optimal timing of initiation, the selection of the most suitable medication, and the appropriate duration of treatment remain controversial (Wat et al., 2019). Furthermore, the irrational utilization of VPA can precipitate a host of avoidable adverse reactions. For instance, as demonstrated by Akyüz et al., it can lead to liver damage, increased drug interactions, skin sensitivities, cognitive impairments, and hinder neurological function recovery (Akyüz et al., 2021), thereby augmenting the economic burden on patients. Consequently, there is an urgent need to validate real-world strategies that ensure the safe and rational administration of this agent.

Structured pharmacist-led interventions using the PDCA (Plan-Do-Check-Act) cycle have been shown to significantly improve the rationality of prescriptions (Hong et al., 2020; Bakshi et al., 2024). This approach is effective in reducing irrational prescriptions, (Bakshi et al., 2024), enhancing medication adherence, (Russell, 2010), and optimizing drug use in various healthcare settings (Yao et al., 2020). For example, a 47% reduction in medication errors was achieved through the systematic application of the PDCA cycle in the emergency department (Salameh et al., 2025). Additionally, in hospital settings, there was a significant decrease in irrational prescriptions, particularly in nonstandard and inappropriate drug use prescriptions (Reis et al., 2023). Moreover, the PDCA cycle has been utilized to enhance the rational use of specific medications like proton pump inhibitors, resulting in a significant decrease in inappropriate prescriptions and associated DDDs (Hong et al., 2020; Chen et al., 2022).

Therefore, implementing structured pharmacist-led interventions using the PDCA cycle may be a promising strategy to address irrational utilization of sodium valproate in patients with neurological disorders. Therefore, we aim to conduct a PDCA cycle to analyze influencing factors based on retrospective data and prospectively evaluate the effectiveness of a structured pharmacist-led intervention program to enhance the rational use of VPA.

## 2 Methods

### 2.1 Study design and setting

**FIGURE 1:**
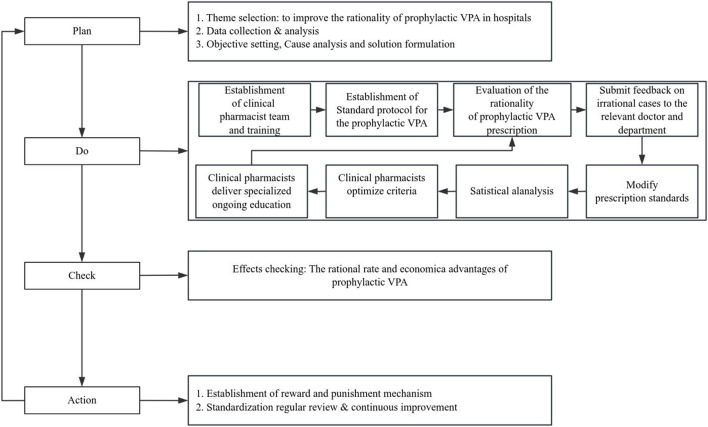
Flow Chart of the Pharmacist-led Intervention Program. This diagram outlines the study design, detailing the sequential stages of the Plan-Do-Check-Act (PDCA) cycle and the integrated eight-step prescription evaluation process used to standardize Sodium Valproate (VPA) administration.

This retrospective pre-post intervention study was carried out in the Department of Neurosurgery at a 1000-bed tertiary hospital in China. The study followed the PDCA cycle to optimize the rational use of VPA (Figure 1). The intervention was structured into five sequential phases spanning from July 2022 to December 2024: Phase I (Baseline assessment, July–December 2022) served as the pre-intervention control, followed by four continuous improvement phases (Phases II–V, January 2023–December 2024).

### 2.2 Participants and data collection

Data were retrieved from hospital’s integrated electronic medical record (EMR) system, focusing on clinical records and administration details for VPA. Patients were included if they were admitted to the Department of Neurosurgery during the study period. Exclusion criteria were defined as: 1) age under 18 years or over 80 years; 2) A documented history of epilepsy or VPA use prior to admission; 3) Mortality occurring during the hospitalization period.

### 2.3 The PDCA intervention framework

#### 2.3.1 Phase 1: PLAN (situation analysis and target setting)

Baseline Data and Evaluation Criteria The baseline “pre-intervention” phase spanned from July 2022 to December 2022. During this period, VPA utilization data—including dosage, duration of therapy, acquisition costs, and DDDs—were collected. A multidisciplinary team (n = 12)—comprising clinical pharmacists, neurologists, an epileptologist, a neurosurgery nurse specialist, and representatives from hospital administration and IT—was established to lead the intervention.

To ensure objective and reproducible assessments of “rational use”, the team first established a standardized clinical pathway and appropriateness criteria for prophylactic VPA in neurosurgery (Supplementary Table S1). Each medication order was assessed against four pillars:

- Indication: Use was restricted to high-risk neurosurgical conditions (e.g., TBI, supratentorial craniotomy) (Supplementary Table S1).
- Administration Route (IV vs. PO): Intravenous (IV) therapy was justified only for patients with impaired consciousness or “nothing by mouth (NPO)” status. Failure to switch to oral (PO) therapy within 24 h of clinical readiness was classified as inappropriate.
- Dosing and Selection: Standard dosing was set at 20–30 mg/kg/day, with adjustments required for hepatic impairment or drug interactions. Deviations without clinical justification were marked as irrational.
- Duration: Prophylaxis was limited to a 3–14 days post-operative window. Continued use beyond 7–14 days in the absence of a seizure event was classified as prolonged duration.

**FIGURE 2:**
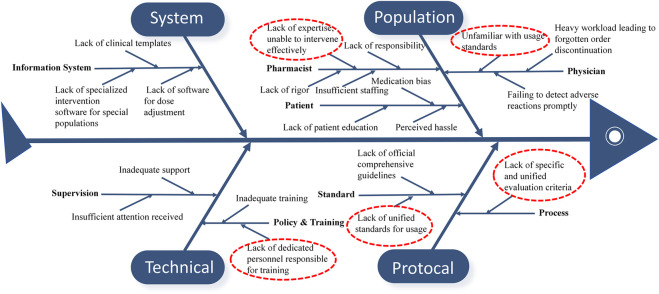
Fishbone (Ishikawa) Diagram Analysis of Factors Contributing to Irrational Sodium Valproate Use. The diagram categorizes the identified root causes of irrational prescribing. Factors are analyzed across six key dimensions: Personnel, Process, Policy, System, Medication, and Patient.

**FIGURE 3:**
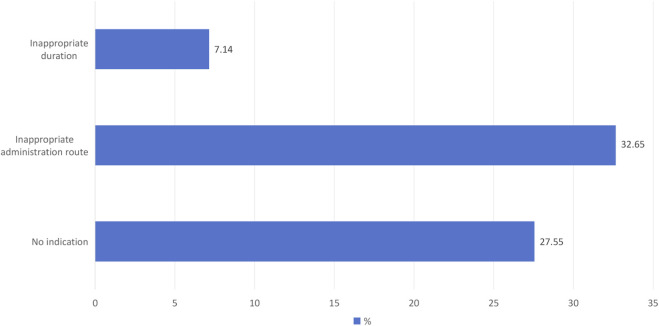
Distribution of Primary Causes for Irrational use of Sodium Valproate during the Baseline Phase (Phase I, N = 98). The bar chart displays a prescription-level analysis of the 66 cases of irrational use identified at baseline. To ensure analytical clarity, each case was assigned a single primary reason for classification, prioritized by the most significant clinical deviation. Note: Classifications were determined through a consensus-based review by two independent clinical pharmacists, with discrepancies adjudicated by a senior clinical pharmacist.

Root Cause Analysis To identify the primary drivers of irrational use, the team applied the Pareto principle (80/20 rule) to baseline data (Figure 2). A fishbone (Ishikawa) diagram was developed to categorize factors across six dimensions: Personnel, Process, Policy, System, Medication, and Patient (Figure 3). The team utilized the “5 Whys” technique to uncover systemic failures, prioritizing factors with a Likert mean score ≥4 intervention.

Target Setting The objective was to evaluate the pharmacist-led intervention’s effect on rationality rates. Target values and compliance rates were established using the following formulas:

Target value=Current Rate+Improved Value

Improved value=Current Rate×Dept. Ratio×Coverage Ratio

- Current Rate: The baseline rate of rational VPA use before intervention.
- Intervention Department Ratio: The proportion of relevant departments receiving intervention. We selected neurosurgery units with the highest prophylactic VPA use, achieving a ratio of 1.0 (100%).
- Intervention Coverage Ratio: The proportion of clinicians within targeted departments receiving education and feedback. This was maintained at 1.0 (100%) through mandatory training.

Target Determination: Using the formula, the initial calculated target was:

$$32\%+\left(32\%\times 1.00\times 1.00\right)=64.00\%$$

Although the formula yielded a target of 64.00%, the multidisciplinary team deemed this insufficient for clinical excellence. Considering feasibility and clinical guidelines, the formal study target was adjusted upward to 90.00%. Success was measured using the Target Compliance Rate, defined as:

$$Target\;Compliance\;Rate=\frac{Completion\;Value\;\left(Actual\;Rate\right)}{Target\;Value\;\left(90\%\right)}\times 100\%$$

- A rate ≥100% indicates the intervention successfully achieved the target.

#### 2.3.2 Phase 2: DO (implementation of countermeasures)

Based on the root cause analysis, four core countermeasures were integrated: (1) standardization of evaluation criteria; (2) clinical education; (3) automated prescription review software; and (4) enhanced supervisory regulations. To clarify the progression of the intervention, the specific “Action” measures implemented in each phase are summarized below:

- Phase I to II (Awareness): Retrospective audits and departmental sessions to highlight prescribing gaps.
- Phase II to III (Standardization): Optimized the clinical operational pathway based on established standard protocols and provided systematic training for medical and nursing staff, emphasizing the necessity of indication assessment before prescribing.
- Phase III to IV (Targeted Feedback): Transition to individualized oversight, where clinical pharmacists performed daily bedside reviews and consultations for “borderline” cases.
- Phase IV to V (Sustainability): Formalization of administrative policies and establishment of a permanent audit-and-feedback mechanism.

#### 2.3.3 Phase 3 & 4: CHECK and ACT (evaluation and improvement)

Prescriptions were assessed according to the predetermined criteria across all phases. To minimize evaluation bias and ensure data integrity, all prescription assessments were conducted independently by two clinical pharmacists. Any discrepancies regarding the “rationality” of a prescription were resolved through consensus-based review, with final adjudication by a senior investigator to ensure consistency in clinical judgment. Statistical outcomes were disseminated to the team biannually, and standardized operational flow charts were updated to reflect these improvements (Figure 1).

### 2.4 Drug utilization and economic metrics

To standardize drug consumption, the Defined Daily Dose (DDD) system (WHO Collaborating Centre) was employed.

- DDD Calculation: Total consumption was determined using the formula:

$$Total\;DDDs=\frac{\text{Total}\;\text{quantity}\;\text{of}\;\text{drug}\;\text{dispensed}\;\left(\text{mg}\right)}{\text{WHO}-\text{assigned}\;\text{DDD}\;\text{value}\;\left(\text{mg}\right)}\times 100\%$$

- Normalization: To facilitate meaningful comparisons across the different PDCA phases despite fluctuations in patient volume, all utilization and economic data were normalized. Metrics are reported as average values per patient-day, utilizing the total LOS as the denominator:

$$Average\;DDDs\;per\;patient-day=\frac{\sum Total\;DDDs\;of\;all\;patients\;in\;phase}{\sum Total\;LOS\;of\;all\;patients\;in\;phase}$$

Economic Assessment Economic outcomes were evaluated based on direct medical costs attributed to VPA (in CNY) using pharmacy procurement prices effective during 2022–2024. Indirect costs (nursing time, consumables) were excluded.

### 2.5 Statistical analysis

Statistical analysis was performed using IBM SPSS software (version 21.0). Categorical variables were presented as frequencies and percentages, while continuous variables were reported as means ± standard deviations (SD). Group comparisons were conducted using Chi-square tests for categorical data and independent t-tests for normally distributed continuous data. The Mann-Whitney U test was applied for non-normally distributed variables. Statistical significance was set at a two-tailed *P*-value <0.05.

## 3 Results

**Patient Characteristics and Baseline Comparability** The PDCA cycle consists of 5 phases, with one phase for pre-PDCA and four phases for post-PDCA and each phase lasting 6 months. The pre-PDCA stage, which spanned from July to December 2022, encompassed 98 patients, while the post-PDCA stages, from January 2023 to December 2024, involved 365 patients. Across all phases, most patients showed no disparities in age, sex, and length of hospital stay, except for a slight increase in hospital stay duration observed in Phase III (Table 1). This indicates a high level of comparability among the groups.

**TABLE 1:** Demographic and clinical Characteristics of neurosurgical patients across pre- and Post-PDCA phases (2022–2024).

Characteristics	Before PDCA	After PDCA			
	Phase I (n = 98)	Phase II (n = 97)	Phase III (n = 102)	Phase IV (n = 76)	Phase V (n = 90)
Male, n (%)	65 (66.3)	70 (72.2)	71 (69.6)	56 (73.7)	62 (68.9)
Age (years)	49.45 ± 15.52	45.80 ± 15.38	46.42 ± 15.28	46.97 ± 14.81	45.73 ± 15.43
Length of stay (days)	16.48 ± 14.69	22.58 ± 25.41	36.37 ± 43.07*	20.43 ± 14.16	18.62 ± 12.32

Study Phases: Phase I (Baseline): July–December 2022; Phase II: January–June 2023; Phase III: July–December 2023; Phase IV: January–June 2024; Phase V: July–December 2024.

Abbreviations: PDCA, Plan-Do-Check-Act.

Statistical Significance: *P < 0.05, **P < 0.01, indicating the significant differences compared to the phase I.

**Improvement in Rational Drug Use** The baseline evaluation (Phase I) revealed a high prevalence of irrational VPA use, with only 32.00% of prescriptions classified as rational. The primary drivers of irrational prescribing were “no indication” (27.55%) and “inappropriate administration route” (32.65%) (Table 2).

**TABLE 2:** Analysis of irrational sodium valproate prescribing patterns and rationality rates pre- and post-intervention.

Item		Pre-PDCA n (%)	Post-PDCA n (%)			
		Phase I (n = 98)	Phase II (n = 97)	Phase III (n = 102)	Phase IV (n = 76)	Phase V (n = 90)
Total rational rate (%)		32.00	51.49^*^	90.99^**^	92.21^**^	93.41^**^
Total irrational rate	No indication	27 (27.55)	22 (22.68)	0 (0.00)	1 (1.32)	0 (0.00)
	Inappropriate administration route	32 (32.65)	21 (21.65)	7 (6.86)	3 (3.95)	2 (2.22)
	Inappropriate duration	7 (7.14)	6 (6.19)	3 (2.94) ^*^	2 (2.63)	4 (4.44) ^**^

Data are presented as frequency n (%). The “Total Rational Rate” represents the percentage of prescriptions fully compliant with the standardized protocol.

Study Phases: Phase I (Baseline): July–December 2022; Phase II–V (Intervention): January 2023–December 2024.

Statistical Significance: *P < 0.05, **P < 0.01, indicating the significant differences compared to the phase I.

**FIGURE 4:**
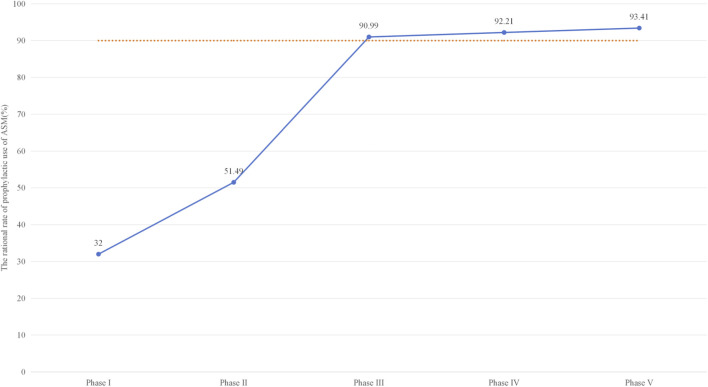
Longitudinal Trends in Rational Sodium Valproate Utilization Across the PDCA Cycle (2022–2024). The trend illustrates the impact of sequential interventions: Phase I–II: Baseline audits and preliminary staff education. Phase II–III: Implementation of the standardized clinical operational pathway. Phase III–IV: Transition to refined, individualized bedside feedback. Phase IV–V: Formal policy institutionalization to ensure long-term sustainability.

To address this issue, we developed a prevention program based on the standard protocol for prophylactic VPA use in different neurosurgical diseases, following reference criteria (Supplementary Table S1). As shown in Table 2 and Figure 4, the structured pharmacist-led intervention significantly improved the overall rational rate of VPA (32% vs. 93.41%), which owe to continues clinical education, and adhere to the daily medical advice review and timely feedback. Compare to Pre-PDCA, no indication, inappropriate administration route, and inappropriate drug use duration were significantly decreased and achieved the most significance in Phase V (27.55%–0.00%, P < 0.01; 32.65%–2.22%, P < 0.01; and 7.14%–4.44%, P < 0.01, respectively).

**Drug Utilization and Economic Outcomes** As shown in Table 3, the implementation of PDCA resulted in a significant reduction in the average duration, cost, and DDDs of injectable VPA. The average duration of injectable VPA decreased from 8.32 ± 6.44 days to 5.71 ± 4.12 days at phase IV and further to 5.37 ± 3.81 days at phase V (P < 0.05). The average cost of injectable VPA per patient - day decreased from 129.70 ± 70.81 CNY to 6.02 ± 5.70 CNY at phase IV and 7.84 ± 8.74 CNY at phase V (P < 0.001). Similarly, the average DDDs of injectable VPA per patient - day decreased from 184.69 ± 50.40 to 12.71 ± 6.51 at phase IV and 17.91 ± 8.92 at phase V (P < 0.05). On the other hand, the average cost of oral VPA per patient - day increased from 0.29 ± 0.74 CNY to 2.04 ± 3.51 CNY at phase IV and 1.64 ± 1.87 CNY at phase V (P < 0.001), while the DDDs of oral VPA per patient - day increased from 0.05 ± 1.12 to 0.46 ± 0.75 at phase IV and 0.39 ± 0.39 at phase V (P < 0.001). The average DDDs of inappropriate injectable VPA decreased from 0.55 ± 0.22 before PDCA to 0.17 ± 0.09 at phase V after PDCA. Overall, the appropriate use of VPA leads to significant cost savings and reduces potential adverse effects.

**TABLE 3:** Impact of the PDCA intervention on the consumption, duration, and economic burden of prophylactic sodium valproate.

Items	Pre-PDCA	Post-PDCA				*P*-value			
	Phase I (n = 98)	Phase II (n = 97)	Phase III (n = 102)	Phase IV (n = 76)	Phase V (n = 90)	P_1_	P_2_	P_3_	P_4_
Duration of injectable VPA (days)	8.32 ± 6.44 11.00 (7.00, 19.00)	12.12 ± 11.10 11.50 (7.75, 22.00)	10.28 ± 7.54 9.50 (4.00, 19.00)	5.71 ± 4.12 4.00 (2.00, 7.00)	5.37 ± 3.81 6.00 (3.00, 8.00)	<0.001	0.056	0.019	0.006
DDDs of injectable VPA per patient - day	0.46 ± 0.26 0.57 (0.47, 0.71)	0.80 ± 0.82 0.58 (0.30, 1.70)	0.44 ± 0.29 0.38 (0.14, 0.61)	0.19 ± 0.31 0.07 (0.05, 0.11)	0.27 ± 0.46 0.08 (0.06, 0.11)	<0.001	0.075	<0.001	0.005
Cost of injectable VPA per patient - day (CNY)	129.70 ± 70.81 159.09 (131.98, 197.97)	232.01 ± 238.59 166.75 (86.48, 490.20)	119.67 ± 90.32 111.61 (38.46, 176.52)	6.02 ± 5.70 1.85 (0.49, 6.58)	7.84 ± 8.74 5.17 (2.98, 7.39)	<0.001	0.560	<0.001	<0.001
DDDs of inappropriate injectable VPA per patient - day	0.55 ± 0.22 0.60 (0.45, 0.71)	0.77 ± 0.73 0.69 (0.48, 0.78)	0.48 ± 0.25 0.52 (0.21, 0.75)	0.23 ± 0.20 0.16 (0.10,0.35)	0.17 ± 0.09 0.16 (0.08, 0.23)	0.016	0.663	0.111	0.058
DDDs of oral VPA per patient - day	0.05 ± 1.12 0.40 (0.30, 0.42)	0.22 ± 0.62 0.68 (0.31.1.26)	0.21 ± 0.52 0.49 (0.14, 0.76)	0.46 ± 0.75 0.33 (0.13.0.52)	0.39 ± 0.39 0.27 (0.14, 0.44)	0.016	0.018	<0.001	<0.001
Cost of oral VPA per patient - day (CNY)	0.29 ± 0.74 2.44 (1.79, 2.56)	0.98 ± 2.95 2.32 (0.76, 6.06)	1.03 ± 2.48 2.37 (0.66, 3.36)	2.04 ± 3.51 1.61 (0.30, 2.51)	1.64 ± 1.87 1.00 (0.30, 2.05)	0.043	0.028	<0.001	0.001

Continuous variables are presented as mean ± standard deviation (SD) and Median (Interquartile Range, IQR) to accurately reflect central tendencies in right-skewed data. Statistical significance was determined by comparing each post-intervention phase to the baseline (Phase I), where:

• P1: Phase II, vs. Phase I.

• P2: Phase III, vs. Phase I.

• P3: Phase IV, vs. Phase I.

• P4: Phase V vs. Phase I.

Study Intervals:

Phase I (Baseline): July 2022 – December 2022.

Phase II (Plan/Do): January 2023 – June 2023.

Phase III (Check/Act): July 2023 – December 2023.

Phase IV (Refinement): January 2024 – June 2024.

Phase V (Institutionalization): July 2024 – December 2024.

Abbreviations: PDCA, Plan-Do-Check-Act; DDDs, Defined Daily Doses; SD, standard deviation; CNY, chinese yuan; VPA, sodium valproate.

**Target Achievement and Continuous Quality Improvement** After the PDCA cycle, the irrational rate of VPA was continually reduced every 6 months. The reasonable rate of VPA was 90.99% in Phase III, which reached the target value of 90% in this study. Compared with pre-PDCA, higher reasonable use of VPA was achieved in Phase V (32.00%–93.41%, P < 0.05). The target compliance rate is 103.79%.

## 4 Discussion

This study demonstrates that a structured, pharmacist-led intervention integrated with the PDCA (Plan-Do-Check-Act) cycle effectively optimizes the rational use of VPA in neurosurgical settings. By establishing a multidisciplinary clinical operational pathway, we achieved a profound shift in prescribing behavior, most notably a significant reduction in “no indication” prescriptions and “inappropriate administration routes”. A critical inflection point was observed in Phase III, with improvements sustained through subsequent phases. This shift was accompanied by tangible stewardship benefits, including a reduction in both the average duration of therapy and the DDDs of injectable VPA. Collectively, these results align with previous research suggesting that active pharmacist involvement is essential for bridging the gap between evidence-based guidelines and daily clinical practice.

Compliance with evidence-based guideline recommendations is crucial for the appropriate utilization of VPA. As a key component of the pharmacist-led intervention program, we have synthesized guideline recommendations and incorporated them with insights from clinical experts to develop a clinical operational pathway for rational use of VPA in different clinical contexts (Liang et al., 2022; Dong et al., 2019). This pathway is disseminated to neurosurgeons through health education and serves as a standard for prescription evaluations. The use of VPA is widespread in neurosurgery but varies significantly depending on the specific condition and perceived risk factors (Wat et al., 2019; Gigliotti et al., 2022; Pease et al., 2024). Although evidence-based guidelines generally discourage routine administration (Walbert et al., 2021; Sayegh et al., 2014)—particularly for brain tumor patients without a history of seizures (Chen et al., 2019) —clinical practice often defaults to prophylaxis. Driven by individual risk assessments and practitioner preference (Siomin et al., 2005), this trend has led to widespread irrational drug utilization. Moreover, adherence to these guidelines in everyday clinical practice has been notably inadequate, partly due to varying levels of comprehension among healthcare providers (Sairanen et al., 2021). Therefore, our evidence synthesis bridges the gap between evidence opinions and clinical scenarios and medical staff awareness, which provides an important approach to ultimately improve the rate of rational drug use.

Pharmacokinetic data indicate that oral VPA demonstrates comparable pharmacokinetic profiles across different administration routes with the bioavailability of oral VPA close to 100% (Nitsche and Mascher, 1982; Hussein et al., 1994). Consequently, intravenous and oral routes present effective alternatives in their respective contexts (Fontana et al., 2020; Ghaleiha et al., 2014). Our investigation revealed problematic practices related to injectable VPA, such as instances of inappropriate duration and no indication, along with delays in transitioning patients to oral medications during hospitalization, resulting in unnecessary extension of intravenous therapy. However, it has been demonstrated that it could effectively maintain therapeutic plasma levels through intravenous loading followed by oral administration (Chen et al., 2025). Promoting timely IV-to-oral conversion remains a critical priority, aligning with the 2021 Chinese National Health Commission measures to mitigate the overuse of intravenous infusions (Sang et al., 2024; Zeng et al., 2019). Furthermore, because the cost of intravenous VPA is typically higher than oral formulations, prompt transitioning represents a cost-effective strategy that reduces both unnecessary expenses and the risks associated with prolonged intravenous access.

The PDCA cycle proved to be a robust framework for continuous quality improvement (Hong et al., 2020; Bakshi et al., 2024). It enabled clinical pharmacists to methodically identify systemic barriers, suggest targeted interventions, and verify implementation effectiveness across multiple phases (Lv et al., 2023). By interconnecting guidelines, medications, pharmacists, and physicians, the PDCA framework allowed for the consistent evaluation of drug selection and duration (Lv et al., 2023; Nguyen et al., 2023; Zhao et al., 2022). However, it is important to clarify that these results reflect improvements in drug utilization metrics and prescribing rationality rather than direct patient-level clinical outcomes (Chen et al., 2022). As this study did not specifically obtain seizure frequency, the incidence of adverse drug reactions, or long-term neurological recovery, the observed “rationality” should be interpreted as a measure of process optimization.

A primary strength of this study lies in its pragmatic design, which successfully integrated the PDCA cycle into the routine clinical workflow. However, as a quasi-experimental before-after study, several threats to internal validity must be acknowledged. First, potential confounding from secular trends in neurosurgical practice, clinician maturation, or subtle shifts in patient case-mix may have influenced the outcomes. Second, comparing multiple sequential post-PDCA phases against a single pre-intervention baseline introduces a risk of inflated Type I error. Furthermore, we did not formally quantify implementation fidelity or account for potential parallel shifts in institutional policy. Future research should employ multi-center, prospective designs to mitigate these biases and provide a more robust evaluation of clinical outcomes.

## 5 Conclusion

In conclusion, the integration of clinical pharmacists into the neurosurgical care team via a PDCA-based framework significantly reduced irrational prescribing, shortened IV treatment durations, and lowered drug costs. These results advocate for the broader adoption of systematic quality improvement methodologies to enhance medication safety and institutional resource management.

## Data Availability

The original contributions presented in the study are included in the article/Supplementary Material, further inquiries can be directed to the corresponding author.
