# Supplementary material for: Enhancing the rational use of sodium valproate in neurosurgery: a pharmacist-led PDCA intervention
Source: Front Pharmacol. 2026 Mar 9;17:1786783. doi: 10.3389/fphar.2026.1786783 (PMC13006234; doi:10.3389/fphar.2026.1786783)
Supplement: Supplementary file 1 [file Table1.docx]

**Supplemental materials**

**eTable 1. Standardized Clinical Operational Pathway and Appropriateness Criteria for Prophylactic Sodium Valproate in Neurosurgery**

**eTable 1. Standardized Clinical Operational Pathway and Appropriateness Criteria for Prophylactic Sodium Valproate in Neurosurgery**

| **Clinical Condition** | **Criteria for Prophylactic Indication** | **Medication & Dosing** | **Administration & Frequency** | **Duration**  **(If Seizure-Free)** | **References** |
| --- | --- | --- | --- | --- | --- |
| Craniocerebral Trauma | 1. Severe craniocerebral trauma, especially open craniocerebral trauma (intracranial abnormal residue and firearm injuries), depressed skull fracture; multiple cortical contusions, intracranial hematoma, and obvious midline shift (>5 mm); 2. Multiple craniocerebral operations; 3. If EEG shows epileptic discharge or is susceptible to seizure (pathogenic gene mutation, family history of seizure, age >40 years, and chronic alcoholism). | VPA: 20–30 mg/kg/day (typically 600–1200 mg/day). | Route: IV or Oral.  Frequency: 2–3 times daily. | Taper and discontinue 7–14 days post-surgery. | ^[1,2]^ |
| Acute Subdural Hematoma | GATE-24 score: ①Glasgow Coma Scale (GCS) 24 hours after operation (GCS score); ②if Anticoagulation: no= 5; ③Timing of operation<24 hours= 5. Epileptic treatment is not required if GATE-24 score =①+②+③≥24 | VPA: 20–30 mg/kg/day (600–1200 mg/day). | Route: IV or Oral.  Frequency: 2–3 times daily. | Discontinue 7–14 days post-surgery. | ^[3]^ |
| Supratentorial Tumors | 1. Incomplete tumor resection; recurrent/progressive tumor; 2. The tumor involves the temporal lobe or motor cortex; the tumor size is large (maximum diameter of the tumor exceeds 3.5–4.5 cm); 3. Susceptibility to seizure: family history of seizures, history of febrile seizures, young age (standards vary between patients with different types of tumors; for example, for glioma, the patient’s age should be <45 years); 4. Long surgery duration (cortical exposure time >4 hour); postoperative imaging examination suggests cerebral infarction; 5. Local placement of slow-release chemotherapeutics or malignant tumors. | VPA: 20–30 mg/kg/day (600–1200 mg/day). | Route: IV or Oral.  Frequency: 2–3 times daily. | Taper and discontinue 14 days post-surgery. | ^[4,2,5]^ |
| Supratentorial Cerebrovascular Disease | 1. Hemorrhagic stroke: the hematoma involves the cortical area; the hematoma volume is large (>10 ml); the cortex is obviously damaged by the operation; EEG reveals epileptic discharge. 2. Intracranial aneurysm: age <40 years, male sex, or middle cerebral artery aneurysm (especially near the medial temporal lobe);  the ruptured aneurysm is bleeding heavily (>15 cm3 of volume of teratoma), or there is loss of consciousness for > 1 h before treatment, Hunt-Hess grade is above grade  III or computed tomography suggests that the Fisher grade is grade III or higher; postoperative imaging examination reveals brain contusion or cerebral infarction. 3.Intracranial arteriovenous malformations: the malformed vascular mass is too large (>4 cm in diameter); it involves the frontal or temporal lobe; it is accompanied by intracranial hemorrhage or local neurological deficits; there is deep vein drainage. 4. Cerebral cavernous vascular malformation: It is recommended that ASMs be routinely preventively used, especially in the case of the following conditions: the malformed vascular mass involves the cortex or medial temporal lobe; lesion diameter >1.5 cm; multiple  lesions; incomplete resection of lesions or band with hemosiderin deposits. | VPA: 20–30 mg/kg/day (600–1200 mg/day). | Route: IV or Oral.  Frequency: 2–3 times daily. | Taper and discontinue 14 days post-surgery. | ^[2]^ |
| Aneurysmal Subarachnoid Hemorrhage (aSAH) | When there are high-risk factors: previous cerebral hemorrhage, cerebral infarction, middle cerebral artery aneurysm rupture, etc. | VPA: 20–30 mg/kg/day (600–1200 mg/day). | Route: IV or Oral.  Frequency: 2–3 times daily. | 3–7 days. | ^[6,7,5]^ |
| Stereotactic Surgery | 1. Deep brain electrode implantation: ASMs are not recommended for routine prophylactic use after deep brain stimulation;53,54 however, intracranial vascular events (hemorrhage, infarction, and edema) during the perioperative period can increase the risk of seizures, and ASMs should be used after surgery. 2. Stereotactic brain biopsy: ASMs are not recommended for routine prophylactic use after stereotactic biopsy;56,57 however, when there are high-risk factors for seizures after  biopsy (there is visible bleeding on imaging and the penetration point is located in the functional area), ASMs can be used prophylactically. | VPA: 20–30 mg/kg/day (600–1200 mg/day). | Route: IV or Oral.  Frequency: 2–3 times daily. | Taper and discontinue 14 days post-surgery. | ^[2]^ |
| Other Cranial Surgeries | Prophylactic use of ASMs would be considered in the following scenarios:   1. Post cranioplasty for cranial defects;   In cases of intracranial abscess or parasitic infestation (particularly when lesions are located in the temporal or parietal lobes, or in patients with extensive cortical damage following craniotomy). | VPA: 20–30 mg/kg/day (600–1200 mg/day). | Route: IV or Oral.  Frequency: 2–3 times daily. | Taper and discontinue 14 days post-surgery. | ^[2, 8]^ |

**General Principles of Application**

1. **Extended Use**: In cases of post-operative intracranial infection or intracerebral hematoma, the duration of VPA application may be extended at clinical discretion.
2. **IV-to-Oral Transition**: Injectable VPA should commence on the day of surgery, typically for 2–3 days. Transition to oral formulations should occur as soon as the patient regains consciousness/tolerates oral intake, with a 12–24 hour overlap to ensure stable therapeutic concentrations.
3. **Monotherapy & Safety**: Monotherapy is preferred for early oral transition. Clinicians should monitor for drug overdose and adverse reactions, particularly during medication overlaps

**Reference:**

1. Liang S, Fan X, Chen F, et al. Chinese guideline on the application of anti-seizure medications in the perioperative period of supratentorial craniocerebral surgery. *Therapeutic advances in neurological disorders* 2022;15:17562864221114357. doi: 10.1177/17562864221114357 [published Online First: 2022/08/23]

2. Dong Y, Guo ZN, Li Q, et al. Chinese Stroke Association guidelines for clinical management of cerebrovascular disorders: executive summary and 2019 update of clinical management of spontaneous subarachnoid haemorrhage. *Stroke and vascular neurology* 2019;4(4):176-81. doi: 10.1136/svn-2019-000296 [published Online First: 2020/02/08]

3. Lanzino G, D'Urso PI, Suarez J. Seizures and anticonvulsants after aneurysmal subarachnoid hemorrhage. *Neurocritical care* 2011;15(2):247-56. doi: 10.1007/s12028-011-9584-x [published Online First: 2011/07/14]

4. Frontera JA, Gilmore EJ, Johnson EL, et al. Guidelines for Seizure Prophylaxis in Adults Hospitalized with Moderate-Severe Traumatic Brain Injury: A Clinical Practice Guideline for Health Care Professionals from the Neurocritical Care Society. *Neurocritical care* 2024;40(3):819-44. doi: 10.1007/s12028-023-01907-x [published Online First: 2024/02/06]

5. Gigliotti MJ, Srikanth S, Cockroft KM. Patterns of prophylactic anticonvulsant use in spontaneous intracerebral and subarachnoid hemorrhage: results of a practitioner survey. *Neurological sciences : official journal of the Italian Neurological Society and of the Italian Society of Clinical Neurophysiology* 2022;43(3):1873-77. doi: 10.1007/s10072-021-05588-2 [published Online First: 2021/09/09]

6. Won SY, Dubinski D, Herrmann E, et al. Epileptic Seizures in Patients Following Surgical Treatment of Acute Subdural Hematoma-Incidence, Risk Factors, Patient Outcome, and Development of New Scoring System for Prophylactic Antiepileptic Treatment (GATE-24 score). *World neurosurgery* 2017;101:416-24. doi: 10.1016/j.wneu.2017.02.024 [published Online First: 2017/02/19]

7. Epilepsy EGoCAA. Expert consensus on the use of antiepileptic drugs after surgery for craniocerebral diseases. *Chinese Journal of Neurosurgery (In Chinese)* 2022;28(07):751-54. doi: 10.3760/cma.j.issn.1001-2346.2012.07.034

8. Qiao-yu W, Zhi-gang Z. Rational prophylactical use of antiepileptic drugs during the perioperative period of neurosurgery: a review. *Clinical Medication Journal* 2018;16(9):9-14. doi: 10.3969/j.issn.1672-3384.2018.09.003
